# Effects of Co-Inoculating *Saccharomyces* spp. with *Bradyrhizobium japonicum* on Atmospheric Nitrogen Fixation in Soybeans (*Glycine max* (L.))

**DOI:** 10.3390/plants12030681

**Published:** 2023-02-03

**Authors:** Obey Kudakwashe Zveushe, Victor Resco de Dios, Hengxing Zhang, Fang Zeng, Siqin Liu, Songrong Shen, Qianlin Kang, Yazhen Zhang, Miao Huang, Ahmed Sarfaraz, Matina Prajapati, Lei Zhou, Wei Zhang, Ying Han, Faqin Dong

**Affiliations:** 1 School of Life Science and Engineering, Southwest University of Science and Technology, Mianyang 621010, China; 2 Department of Crop and Forest Sciences, University of Lleida, 25198 Lleida, Spain; 3 Joint Research Unit CTFC-AGROTECNIO, Universitat de Lleida, 25198 Lleida, Spain; 4 Fundamental Science on Nuclear Wastes and Environmental Safety Laboratory, Southwest University of Science and Technology, Mianyang 621010, China; 5 Center of Analysis and Testing, Southwest University of Science and Technology, Mianyang 621010, China; 6 School of Environment and Resource, Southwest University of Science and Technology, Mianyang 621010, China; 7 Key Laboratory of Solid Waste Treatment and Resource Recycle, Southwest University of Science and Technology, Mianyang 621010, China

**Keywords:** co-inoculation, nitrogen fixation rate, soybean, *Saccharomyces cerevisiae*, *Saccharomyces exiguus*, *Bradyrhizobium japonicum*, phosphorus solubilization, Idole-3-acetic acid/auxin (IAA)

## Abstract

Crop production encounters challenges due to the dearth of nitrogen (N) and phosphorus (P), while excessive chemical fertilizer use causes environmental hazards. The use of N-fixing microbes and P-solubilizing microbes (PSMs) can be a sustainable strategy to overcome these problems. Here, we conducted a greenhouse pot experiment following a completely randomized blocked design to elucidate the influence of co-inoculating N-fixing bacteria (*Bradyrhizobium japonicum*) and PSMs (*Saccharomyces cerevisiae* and *Saccharomyces exiguus*) on atmospheric N_2_-fixation, growth, and yield. The results indicate a significant influence of interaction on Indole-3-acetic acid production, P solubilization, seedling germination, and growth. It was also found that atmospheric N_2_-fixation, nodule number per plant, nodule dry weight, straw, and root dry weight per plant at different growth stages were significantly increased under dual inoculation treatments relative to single inoculation or no inoculation treatment. Increased seed yield and N and P accumulation were also noticed under co-inoculation treatments. Soil available N was highest under sole bacterial inoculation and lowest under the control treatment, while soil available P was highest under co-inoculation treatments and lowest under the control treatment. We demonstrated that the co-inoculation of N-fixing bacteria and PSMs enhances P bioavailability and atmospheric N_2_-fixation in soybeans leading to improved soil fertility, raising crop yields, and promoting sustainable agriculture.

## 1. Introduction

Phosphorus (P) and nitrogen (N) are essential macronutrients for soybean [*Glycine max* (L.) Merrill] growth and function. Soybean is a crop of great importance in human and livestock nutrition and requires large amounts of N and P to attain high-quality yields. Historical gains in soybean seed yields are primarily due to increases in seed biomass, which demonstrate an improvement in seed partitioning efficiency [1]. Worldwide, agricultural activity is limited by P and N. The increase in partitioning efficiency and seed biomass require larger N demand [2], primarily met by biological di-nitrogen (N_2_)-fixation (BNF) and soil N mineralization. 

Rock phosphate is a relatively inexpensive source of P, and therefore, widely used as a fertilizer. However, there are numerous limitations related to its use. For instance, it is generally well known that P is not readily available from rock phosphate because the solubility of phosphate is low and it forms insoluble complexes with soil iron (Fe), aluminum (Al), and calcium (Ca) [3,4]. Several researchers also reported a decline in soybean growth and metabolism due to P deficiency [5,6], while Onor et al. and Chiluwal et al. [7,8] reported a decline in soybean productivity with low N nutrition. To satisfy crop nutritional requirements, P and N are usually added to the soil as chemical P or N fertilizers; synthesis and continuous addition of any of these fertilizers is a highly energy-intensive process. The world demand for P and N has gradually increased over the past decades, but rock phosphate addition is non-sustainable and currently achieving alarming levels considering available finite global rock phosphate reserves [9]. In addition, N is one of the most insufficient mineral elements in the soil. The above problems are exacerbated by the vulnerability of soil P to immobilization and the high mobility state of N. An estimated 78–79% N is available in the atmosphere in inert structure (N_2_) that is not available for direct plant absorption, making only about 20% of N directly available for plant absorption; this is a very small amount considering the amount of N required by a plant for growth and development [10]. Moreover, plants can use only a small amount of this P since 75–90% of added P is precipitated by metal-cation complexes and is quickly fixed in the soil [11,12]. Finding solutions to the inefficiency of N and P fertilization is a crucial unsolved problem that has been under investigation for more than half a century [13]. It is important to find sustainable solutions to increase N and P fertilization and this could be achieved by improving the BNF efficiency of nitrifying bacteria and P solubilization by phosphate-solubilizing microorganisms (PSM) to reduce the continuous application of chemical fertilizers in the soil. Bio-fertilizers/bio inoculants are composed of beneficial microorganisms: they are low-cost, effective, and renewable sources of plant nutrients to supplement chemical fertilizers. In addition to their role in enhancing the growth of plants, they can act as bio-control agents in the rhizosphere at the same time. P bio-fertilizers (PSMs) make P accessible for plant absorption by applying various approaches such as lowering soil PH, chelation, and mineralization [6,10]. They also promote plant growth through the production of plant growth-promoting substances and by elevating the efficiency of BNF [14]. As N and P are major plant nutrients and combined inoculation of BNFs and PSM may benefit plants better than either group of organisms in solo, this synergistic effect, when present, enhances the role of applying bio-fertilizers in the sustainable agriculture setup while mitigating the increasing cost of fertilizers and their impact on the environment. 

BNF by the symbiotic association between the plant and soil bacteria (mainly from the genus *Bradyrhizobium*) is regarded as the least expensive and most efficient source of nitrogen for soybeans. Since the efficiency of the BNF depends on factors such as bacterial species and strain, crop cultivar, symbiosis specificity, and prevailing environment [15,16,17], bacteria that form a symbiosis with soybean nodules are *Bradyrhizobium japonicum*, *Bradyrhizobium elkani,* and *Sinorhizobium fredii*. In this community, approximately 20–400 kg/ha/annum of nitrogen is fixed and transferred to the plant, reducing the need for synthetic nitrogen fertilizer use [18]. Studies have found that some other microorganisms in the soil can affect the vitality of the nitrifying bacteria, which in turn affects its N_2_-fixation efficiency [19,20]. Improving bacterial N_2_-fixation in grain legumes is pivotal to the sustainable intensification of agriculture. Several studies have reported the limitation of N_2_-fixation in legumes due to P deficiency, particularly those growing in acid tropical and subtropical soils [9,21]. Studies have also shown that providing relevant microbial symbionts for plants can optimize the successful supplementation of soil nutrition to plants. Although the relevance of biological nitrogen nutrition of soybeans [*Glycine max* (L.) Merr.] is recognized worldwide, inoculation with *B. japonicum* shows variable results and requires supplementation by other synergistic microbes to offer further support to the crop under production [22,23]. The measurement of BNF in legumes is essential for deriving a profound knowledge of their contributions to the N economies of agricultural systems and for N management in agricultural systems [24], and so is the measurement of the effect of soil yeast on N_2_-fixation and plant productivity.

Understudied, yet potentially important microbes in the area of plant nutrition are yeasts such as those from the *Saccharomyces* genera. Some *Saccharomyces* microbes can increase the availability of nutrient elements such as P, iron (Fe), and Calcium (Ca) through their biological activities [19,25,26,27]. The can also help to improve soil health and may influence plant growth indirectly by encouraging the growth of other plant growth-promoting rhizo-microorganisms through the production of plant growth hormones such as indole-3-acetic acid/auxins (IAA), polyamines, vitamin B_12,_ protein, organic acids, soluble, and volatile exudates as well as solubilizing inorganic P and iron [19,26,28,29]. These PSMs have also been reported to enhance N_2_-fixation, nodulation, root growth, nutrient uptake, and disease tolerance in different plants [30,31,32,33]. Furthermore, some studies have shown that dual inoculation of these plant growth-promoting microbes improves plant growth relative to the sole inoculation of only one [34,35]. For instance, Shome et al. [34] reported an increase in the growth, yield, and quality of soybean after inoculating the soybean seeds with *Rhizobium* and P-solubilizing bacteria. Kumawat et al. [36] also reported that *Rhizobium* sp. LSMR-32 and *Enterococcus mundtii* LSMRS-3 simultaneously induce salt tolerance and improve productivity in spring mung bean under saline stress conditions.

To increase BNF in soybeans and seed yield, we compared the performance of the BNF process in soybeans and the performance of soybeans under no microbial inoculation, single inoculation, or dual microbial inoculation. Before inoculating the seeds of the soybean with the microbes, we first performed in vitro experiments to determine if there is any form of antagonism among the microbes, and also to determine the capability of each microbial strain to produce IAA and solubilize P. After growing soybeans under different microbial inoculations (refer to the materials and methods section for the microbial treatments), we determined the interacting effects of bacterial and yeast inoculation on seed germination and seedling growth, i.e., shoot and root length, and seedling vigor index (SVI). We then determined the number of nodules per plant (NNP), nodule dry weight per plant (NDWP), and the BNF in soybeans by measuring the concentration percentage of N derived from the atmosphere (%Ndfa), soybean growth (straw and root dry weights, nodule dry weight, nodule), and N and P accumulation in soybean straw and seeds. In particular, we hypothesized that (1) the BNF is higher in dual inoculated soybeans than single inoculated soybeans; (2) seed yield is higher in dual inoculated soybeans relative to single inoculated soybeans; (3) accumulation of P and N in seeds and straw is higher in dual inoculated soybeans than single inoculated soybeans. Our study is the first aimed at elucidating the influence of nitrifying bacteria and P-solubilizing yeast on the actual %Ndfa using the stem bleeding technique in soybeans.

## 2. Results

### 2.1. Inorganic Phosphate Solubilization and Solubilization Index by the Microbial Isolates

**Figure 1:**
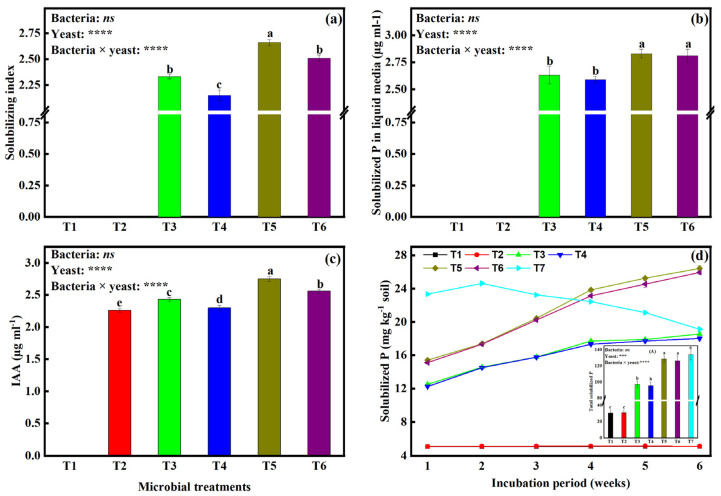
In vitro measurements of (**a**) P solubilization index, (**b**) solubilized P in liquid media, (**c**) IAA production, and (**d**) solubilized P in soil under different microbial treatments. Data (means ± SE, *n* = 5). *p*-values of two-way ANOVAs followed by different letters denote significant differences between treatments at *p* < 0.05 according to Tukey’s HSD. *p*-values of two-way ANOVAs of bacteria, yeast, and their interaction (bacteria × yeast) are indicated: *p* < 0.05, *; *p* < 0.01, **; *p* < 0.001, ***; *p* < 0.0001, ****; ns, not significant. Treatments: non-inoculated soybeans (T1), soybean inoculated with *B. japonicum* (T2), *S. cerevisiae* (T3), *S. exiguus* (T4), *B. japonicum* × *S. cerevisiae* (T5), *B. japonicum* × *S. exiguus* (T6), or non-inoculated soil with the addition of 2 mL of 0.1 M KH_2_PO_4_·2H_2_O (T7).

To check for potential antagonism between our microbial isolates, we co-cultured bacteria and yeast. Our results indicate that bacteria could coexist with any of the two yeast isolates (Figure S1a). We observed that data showed different diameters of the clear zone of phosphate dissolution on Pikovskaya agar medium (Figure 1a and Figure S1b). The phosphate solubilization index (SI) was 0 under treatments T1 and T2. We also found that the treatments that contained yeast isolates in them managed to solubilize P. The SI was highest under treatment T5 (2.66), followed by T6 (2.51), then T3 (2.33), and T4 had the lowest SI (2.15). The SI values were significantly different from each other across all treatments. Furthermore, the strains were also studied for their ability to solubilize phosphorus from insoluble phosphate tricalcium phosphate (Ca_3_(PO_4_)_2_) in Pikovskaya liquid media. Levels of P solubilized from Ca_3_(PO_4_)_2_ by the microbial isolates under different combinations are shown in Figure 1b. Our data indicate that treatment T5 produced the highest amounts of solubilized P (2.6 μg mL^−1^), followed by T6 (2.81 μg mL^−1^), then T3 (2.63), and T4 with the lowest values (2.59). We noticed an insignificant difference between T3 and T4, and also between T5 and T6. 

The same treatment combinations also showed varied abilities to solubilize inorganic phosphates in soil media after weeks one to six of incubation (Figure 1d). The experiment showed that one week after the commencement of the incubation, all the treatments which included yeast isolates in them (*S. cerevisiae, S. exiguus, B. japonicum × S. cerevisiae,* and *B. japonicum × S. exiguus*) showed significantly higher values of P solubilization (over 5.2 mg kg^−1^ soil) than T1 and T2, (non-inoculated and inoculated with *B. japonicum*, respectively). On the one hand, weekly measurements of solubilized P during the incubation period showed that solubilized P quantities were significantly higher under treatments with both bacteria and any one of the yeast isolates (dual inoculants or co-inoculants, hereafter) (ranging from 15.12 mg P kg^−1^ to 26.42 mg P kg^−1^ soil). On the other hand, treatments that only contained one of the two different yeast isolates solubilized from 12.26 mg P kg^−1^ to 18.01 mg P kg^−1^ (range). There was also an insignificant amount of P solubilized between treatment T3 and T4 and also between T5 and T6.

### 2.2. In Vitro Determination of IAA Production in Liquid Media by Different Microbial Isolates

We assessed the potentialities of the microbial isolates to in vitro produce the phytohormone (IAA) under different isolate combinations (Figure 1c). After five days of culturing, treatments T2 (*B. japonicum*), T3 (*S. cerevisiae* alone), and T4 (*S. exiguus* alone) produced 2.26, 2.43, and 2.3 μg/mL of IAA, respectively, while T5 (*B. japonicum* × *S. cerevisiae*) and T6 (*B. japonicum* × *S. exiguus*) had produced significantly higher quantities of IAA (2.75 μg/mL and 2.56 μg/mL, respectively) relative to T3 and T4. Furthermore, we could not detect any IAA production under treatments T1 and T2.

### 2.3. Soybean Seedling Growth and Development under Different Microbial Treatments

We observed significant and interactive effects of bacteria inoculation and yeast inoculation on seedling vigor index (SVI) (*p* < 0.001, Table 1). SVI was highest in seedlings under dual inoculation treatments at 32.2 (T5) and 31.5 (T6). The average SVI for all other treatments (T1, T2, T3, and T4) ranged from 17.9 to 24.6. It is also worth mentioning that there was an insignificant difference in SVI between T5 and T6, and also between T3 and T4.

There were insignificant interactive effects of bacterial inoculation and yeast inoculation on seedling-specific root length (SRL) and germination percentage (GP) (*p* > 0.5, Table 1). SRL was 0.03 across all the treatments, while Gp ranged between 97 and 98% across the treatments.

**Figure 2:**
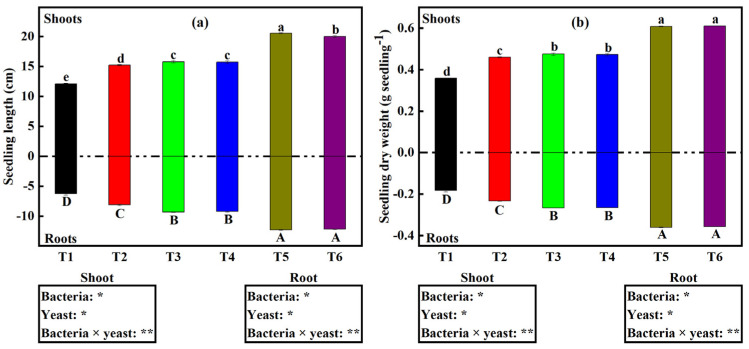
(**a**) Shoot and root length (**b**) shoot and root dry weight, of soybean seedlings under different microbial treatments. Data (means ± SE, *n* = 5). Bars with lowercase letters and uppercase letters denote shoots and roots, respectively. *p*-values of two-way ANOVAs followed by different letters denote significant differences between treatments at *p* < 0.05 according to Tukey’s HSD. *p*-values of two-way ANOVAs of bacteria, yeast, and their interaction (bacteria × yeast) are indicated: *p* < 0.05, *; *p* < 0.01, **; *p* < 0.001, ***; *p* < 0.0001, ****; ns, not significant. Treatments: non-inoculated soybeans (T1), soybean inoculated with *B. japonicum* (T2), *S. cerevisiae* (T3), *S. exiguus* (T4), *B. japonicum* × *S. cerevisiae* (T5), or *B. japonicum* × *S. exiguus* (T6).

The shoot length and root length of seedlings varied across treatments (Figure 2a). On the one hand, seedling shoot length was significantly higher under treatments T5 (20.6 cm) and T6 (20 cm). The average seedling shoot length for all other treatments (T1, T2, T3, and T4) ranged from 12.1 to 15.8 cm. We noticed an insignificant difference only between treatments T3 and T4. On the other hand, seedling root length was significantly higher under treatments T5 (12.3 cm) and T6 (12.2 cm). The average seedling root length for all the treatments (T1, T2, T3, and T4) ranged from 6.3 to 9.3 cm. Unlike for seedling shoot length, we found insignificant differences in seedling root length between T5 and T6, and also between T3 and T4 (Figure 2a).

Bacteria and yeast inoculation also showed a significant (*p* < 0.01) interactive effect over the seedling shoot dry weight (SSDW) and seedling root dry weight (SRDW) (Figure 2b). Seedlings under both dual inoculations showed significantly higher SRDW and SSDW (0.61 g seedling^−1^ and 0.36 g seedling^−1^, respectively). Average SSDW and SRDW for the sole inoculations and the control ranged between 0.36 and 0.61 g seedling^−1^ (shoots) and 0.18 to 0.36 g seedling^−1^ (roots). There was no significant difference between T3 and T4, and also between T5 and T6.

**Figure 3:**
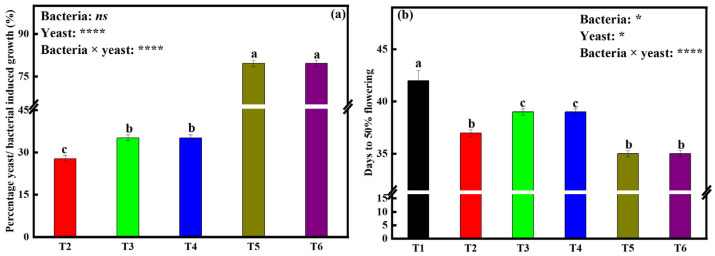
Measurements of (**a**) percentage yeast/bacterial-induced growth and (**b**) days taken to reach 50% flowering of soybean seedlings under different microbial treatments. Data (means ± SE, *n* = 5). *p*-values of two-way ANOVAs followed by different letters denote significant differences between treatments at *p* < 0.05 according to Tukey’s HSD. *p*-values of two-way ANOVAs of bacteria, yeast, and their interaction (bacteria × yeast) are indicated: *p* < 0.05, *; *p* < 0.01, **; *p* < 0.001, ***; *p* < 0.0001, ****; ns, not significant. Treatments: non-inoculated soybeans (T1), soybean inoculated with *B. japonicum* (T2), *S. cerevisiae* (T3), *S. exiguus* (T4), *B. japonicum* × *S. cerevisiae* (T5), or *B. japonicum* × *S. exiguus* (T6).

To understand the percentage influence of each microbial treatment on soybean growth, we assessed the seedling growth that is a result of (1) Bacterial inoculation (bacterial-induced growth response); (2) Yeast inoculation (yeast-induced root growth response); and (3) The combination of bacteria and yeast. We noticed significant (*p* < 0.001) and interactive effects of bacterial and yeast co-inoculation on the percentage microbial-induced root growth response (Figure 3a). Sole yeast inoculation (T3 and T4) led to a significant increase in induced growth (35.2% and 35.1%, respectively) relative to the sole bacterial inoculation treatment T2 (27.8%). Furthermore, dual inoculation (T5 and T6) led to a significantly higher percentage induced growth response of 79.6% for both treatments. We noticed insignificant microbial-induced growth responses between treatments T3 and T4, and also between T5 and T6. 

### 2.4. Soybean Root-Nodulation-Related Traits under Different Microbial Treatments

Root nodule number per plant (NNP) and nodule dry weight per plant (NDWP) were measured at V_5_ and R_3.5_ physiological stages. Our results indicate that the bacteria-yeast interaction significantly (*p* < 0.001) affected the NNP and NDWP (Table S3). We noticed an increase in NNP from the V_5_ to the R_3.5_ stage. The NNP was highest on soybean roots under dual inoculation treatments T5 (36.8 and 78.6, V_5_ to R_3.5_, respectively), and T6 (33.8 and 74.3, V_5_ to R_3.5_, respectively). Average NNP under sole inoculation treatments ranged from 19.5 to 26.5 (V_5_) and from 27.8 to 33; R_3.5_ which was significantly lower than NNP under dual inoculation but significantly higher than those under the control treatment ranged between 13.3 V_5_ and 14.5 R_3.5_. There was also an insignificant difference between T3 and T4 and also between T5 and T6 (Table S3). The NDWP also followed the same trend as NNP (Table S3). The NDWP was highest under dual inoculation treatments ranging from 580.7 to 591.3 mg plant^−1^ and 1259.3 mg plant^−1^ to 1277.3 (V_5_ to R_3.5_, respectively). Average NDWP under sole inoculation treatments had intermediate values ranging from 336.3 to 430.5 plant^−1^ and 468.5 to 536.3 plant^−1^ (V_5_ to R_3.5_, respectively). There was also an insignificant difference between T3 and T4 and also between T5 and T6. 

### 2.5. Atmospheric N_2_-Fixation in Soybeans under Different Microbial Treatments

In terms of N_2_-fixation, we observed significant and interactive (*p* < 0.001) differences in relative ureide-N% (RUN%) and %Ndfa across the treatments at both V_5_ and R_3.5_ stages (Table 2). RUN% values were highest in the xylem sap of dual inoculated soybeans, namely T5 (61.3% and 77.8%, V_5_ and R_3.5_, respectively) and T6 (58.2% and 75%, V_5_ and R_3.5_, respectively). Single-inoculated soybeans had significantly lower percentage values relative to the dual-inoculated soybeans, but significantly higher relative to the control treatment (from 6.5% to 50.1% V_5_ range and from 10.3% to 62.3%). The lowest RUN% values were in soybeans under the control treatment (5.4% and 9.4%, V_5_ and R_3.5_, respectively). We also found insignificant differences between treatments T5 and T6, and also between treatments T3 and T4).

As for %Ndfa%, the N_2_-fixation only occurred under treatment T2 (66.2% and 72.4%, V_5,_ and R_3.5_, respectively), T5 (83.7% and 90.7%, V_5_ and R_3.5_, respectively), and T6 (78.8% and 88.3%, V_5_ and R_3.5_, respectively) (Table 2). Insignificant differences were noticed between treatments T5 and T6, and also between treatments T3 and T4. 

### 2.6. Effects of Bacterial and Yeast Inoculation on Time Taken to Reach 50% Flowering

We found significant (*p* < 0.001) and interactive effects of bacteria and yeast inoculation on time (days) taken by soybean plants to reach 50% flowering (Figure 3b). The number of days taken to reach 50% flowering was lowest on soybeans under treatments T5 and T6 (35), followed by T2 (37), then T3 and T4 (39). Soybeans under the control treatment had the highest number of days (42). There was no significant difference between treatment T3 and T4, and also between treatment T5 and T6.

### 2.7. Effects of Bacterial and Yeast Inoculation on Seed Number per Plant (SNP) and Pod Number per Plant (PNP)

**Figure 4:**
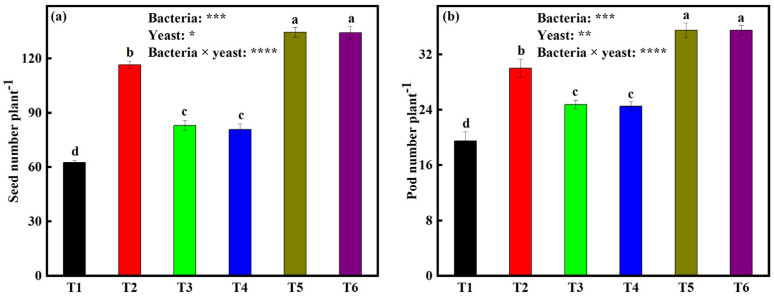
Measurements of (**a**) seed number plant^−1^ and (**b**) pod number plant^−1^ of soybean seedlings under different microbial treatments at the R_8_ physiological stage. Data (means ± SE, *n* = 5). *p*-values of two-way ANOVAs followed by different letters denote significant differences between treatments at *p* < 0.05 according to Tukey’s HSD. *p*-values of two-way ANOVAs of bacteria, yeast, and their interaction (bacteria × yeast) are indicated: *p* < 0.05, *; *p* < 0.01, **; *p* < 0.001, ***; *p* < 0.0001, ****; ns, not significant. Treatments: non-inoculated soybeans (T1), soybean inoculated with *B. japonicum* (T2), *S. cerevisiae* (T3), *S. exiguus* (T4), *B. japonicum* × *S. cerevisiae* (T5), or *B. japonicum* × *S. exiguus* (T6).

We observed that SNP was significantly influenced (*p* < 0.001) by the interactive effects of bacteria and yeast inoculation (Figure 4a). After harvesting (R_8_ stage), we found that SNP was highest on soybeans under dual inoculation. Treatments T5 and T6 led to a 115.2% and 114.8% increase in SNP when compared to the control treatment (62.5 SNP). As for single inoculation treatments, all of the soybean plants under the single inoculation treatments had significantly lower NSP relative to dual inoculated soybeans. However, single-inoculated soybeans had significantly higher NSP (average range from 29.2% to 85.6%) relative to soybeans under the control treatment. Furthermore, we did not find a significant difference between T3 and T4, and also between T5 and T6. 

PNP after harvesting (R_8_ stage) followed a similar trend as with NSP. NSP was also significantly (*p* < 0.001) influenced by the interaction effects of bacteria and yeast inoculation (Figure 4b). PNP was highest on soybeans under treatment T5 and T6, both with an 82% increase in PNP relative to the control treatment (19.5, PNP). We also noticed a significant increase in PNP on soybeans under single inoculations with an average range from 25.6 to 53.8% relative to the control. Furthermore, we did not find a significant difference between T3 and T4, and also between T5 and T6. Furthermore, we did not find a significant difference between T3 and T4, and also between T5 and T6.

### 2.8. Straw and Seed N and P Concentrations under Different Microbial Treatments 

Straw concentrations of N and P at the R_8_ stage were significantly affected by the interaction between bacteria and yeast isolates (*p* < 0.001) (Table 3). N concentrations in shoots increased with microbial inoculation. To be specific, N concentration in shoots under treatment T1 was 28.51 mg g^−1^. Dual inoculation (T5 and T6) led to an increase in straw N concentration with 142.7 and 135.8%, respectively, relative to T1. We also noticed an average increase of 30.7 to 106% in straw N concentration under single inoculations relative to T1. It is worth mentioning that there was no significant difference in straw N concentrations between T3 and T4, and also between T5 and T6.

Straw P concentrations also followed the same trend as straw N concentrations (Table 3). Specifically, the lowest P concentrations were found in straw under treatment T1 (72.91 mg g^−1^). Relative to T1, the highest increase in straw P concentration was under dual inoculations T5 (66.9%) and T6 (76.1%). Increases in straw P concentration under single inoculations ranged from 10.8 to 33.4% relative to T1. Insignificant differences in straw P concentrations were noticed between treatments T3 and T4, and also between treatments T5 and T6. 

### 2.9. Influence of Bacteria and Yeast Inoculation on Seed N and P Concentrations, and Rhizosphere Available P Concentration

Seed N and P concentrations at the R_8_ stage were significantly influenced by the interaction of bacteria and yeast (Table 3). The lowest seed N concentration was found under T1 (50.57 mg g^−1^). Relative to the T1, the highest seed N concentrations were under dual inoculations T5 (68.7%) and T6 (68%). Increases in seed N concentration under single inoculations ranged from 25.4 to 50% relative to T1. It is worth mentioning that there was no significant difference in seed N concentrations between T3 and T4, and also between T5 and T6.

Seed P concentrations also followed the same trend as seed N concentrations (Table 3). The lowest P concentrations were found under treatment T1 (102.91 mg g^−1^). Relative to T1, seed P concentrations under dual inoculations increased by 47.4 and 44.2% for T5 and T6, respectively. Furthermore, the increase in seed N concentration under single inoculations ranged from 10.5 to 19.7% relative to T1. There were no significant differences in seed P concentrations between treatment T3 and T4, and also between T5 and T6. 

### 2.10. Influence of Bacterial and Yeast Inoculation on Straw Dry Weight (SDW)

**Figure 5:**
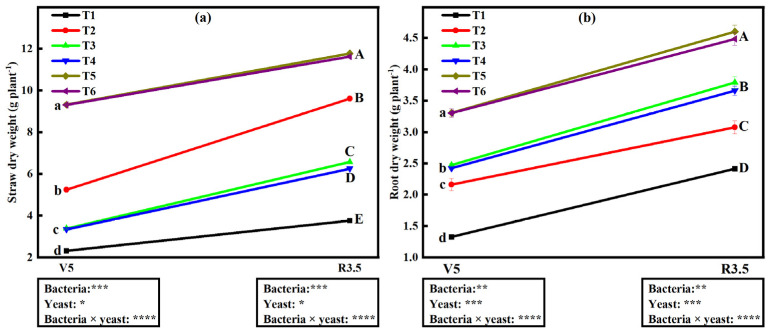
Measurements of (**a**) straw dry weight and (**b**) root dry weight of soybean straw under different microbial treatments at V_5_ and R_8_ physiological stages. Data (means ± SE, *n* = 5). Points with lowercase letters and uppercase letters denote V_5_ stage and R_3.5_, respectively. *p*-values of two-way ANOVAs followed by different letters denote significant differences between treatments at *p* < 0.05 in the same stage according to Tukey’s HSD. *p*-values of two-way ANOVAs of bacteria, yeast, and their interaction (bacteria × yeast) are indicated: *p* < 0.05, *; *p* < 0.01, **; *p* < 0.001, ***; *p* < 0.0001, ****; ns, not significant. Treatments: non-inoculated soybeans (T1), soybean inoculated with *B. japonicum* (T2), *S. cerevisiae* (T3), *S. exiguus* (T4), *B. japonicum* × *S. cerevisiae* (T5), or *B. japonicum* × *S. exiguus* (T6).

The ANOVA results showed significant and interactive effects on straw dry weight SDW (*p* < 0.0001) at both V_5_ and R_8_ stages (Figure 5a). The lowest SDW was 2.31 g plant^−1^ V_5_ and 3.76 g plant^−1^ R_8_. We also noticed that SDW was highest under dual inoculation increasing by 303.5 and 303% for T5 and T6, respectively, at the V_5_ stage, and 213.3 and 209.3% at R_8_ compared to T1. Furthermore, SDW ranged from 66.2 to 155.6% under single inoculations relative to T1. Insignificant differences in SDW were noticed between treatments T3 and T4 and also between treatments T5 and T6 at the V5 stage, and only between T5 and T6 at the R_8_ stage.

### 2.11. Influence of Bacterial and Yeast Inoculation on Root Dry Weight (RDW)

Results showed significant (*p* < 0.0001) and interactive influence of bacteria and yeast inoculation on RDW at both V_5_ and R_8_ stages (Figure 5b). The lowest RDW values were found on soybeans grown under T1 (1.33 g plant^−1^ and 2.41 g plant^−1^, V_5,_ and R_8_, respectively). The RDW under dual inoculation treatments increased by 149.6 and 149% for T5 and T6, respectively, at the V_5_ stage, and 90.8 and 85.9% at the R_8_ stage relative to T1. Furthermore, RDW ranged from 63.2 to 86.6% at V_5_ and from 27.5 to 57.1% at R_8_ under single inoculations relative to T1. Significant differences in RDW were found between T3 and T4, and also between T5 and T6 at both V_5_ and R_3.5_ stages.

### 2.12. Influence of Bacteria and Yeast Inoculation on Rhizosphere Soil Available N and P Concentration

Our experiment result showed significant interaction effects of bacteria and yeast co-inoculation on N (*p* < 0.01) and P (*p* < 0.001) concentrations in the rhizosphere soil (Table S4). Available N in soils under T1 was 5.52 mg g^−1^ soil. We noticed an increase in the soil available N under single inoculations relative to treatment T1 in a range from 3.8 to 12.5%. However, declines of 3.4 (T3) and 3.9% (T4) in available soil N were also observed relative to T1. On the other hand, in contrast to straw and seed P concentrations, available P was lowest under treatment T2, which was 7.5% lower than that under the control treatment (T1). We also noticed a 71.1, 69.9, 85.5, and 85% increase in available P (T1, T3, T4, T5, and T6, respectively), relative to T2. Furthermore, a 59.1, 58.1, 72.6, and 72% increase in rhizosphere available P (T3, T4, T5, and T6, respectively) was noticed relative to T1. However, insignificant differences were noticed between treatments T3 and T4, and also between treatments T5 and T6 for both available N and P concentrations.

## 3. Discussion

### 3.1. Co-Culturing of Bacteria and Yeast Enhances Inorganic P Solubilization and IAA Production by Yeast

The ability of microbes to solubilize inorganic P is one of the crucial traits that qualify several to be harnessed for sustainable crop production. The following are the results from this experiment: (1) Bacteria alone could not solubilize inorganic P in both liquid and on solid (agar and soil) media, but could produce considerable amounts of IAA; (2) Yeast alone could solubilize considerable amounts of inorganic P and also produce considerable amounts of IAA; but furthermore, (3) Co-culturing of bacteria and any of the two yeast strains led to even higher amounts of inorganic P to be solubilized and IAA produced (Figure 1a,b,d and Figure S1). Although the reason for higher values of solubilized P and IAA produced is not clear, we postulate that it is because of the interaction between bacteria and yeast. Therefore, we suggest that more research should be completed to understand how co-culturing of IAA producing and P solubilizing yeasts lead to both improved IAA production and P solubilization.

### 3.2. Dual Inoculation of Bacteria and Yeast Enhances Soybean Seedling Establishment

Seedling establishment is one of the critical stages in a plant’s life cycle, contributing to the increased yield and quality of cultivated crop plants [37]. Based on our experiment’s results, we found that soybean seedling shoot and root lengths and dry weight were higher under treatments that contained a yeast isolate, and dual inoculation of bacteria and yeast led to even higher levels than all single inoculation treatments (Figure 2). Numerous studies have revealed that yeast inoculation on seeds enhances seed germination. These results are in line with those reported by [38,39], who found that yeast genera *Cryptococcus* and *Rhodotorula* were able to solubilize low soluble phosphorus sources and accumulate polyphosphates, and also affected root growth. Based on the study results confirming IAA production by both bacteria and yeast isolates in our experiment (Figure 1c) and considering IAA to be one of the growth regulators produced (IAA is notoriously known for promoting apical meristematic growth) [40], this might also one of the reasons why soybean seedlings growth under T3, T4, T5, and T6 were higher relative to T1 and T2. Additionally, since our experiment of co-culturing bacteria and yeast isolates led to higher IAA production, this might also explain the higher soybean seedling shoot length and dry weight and also root length and dry weight under treatment T5 and T6.

Growth parameters such as SRL and SVI (Table 1), %YGR, and %BGR (Figure 3a) were also significantly influenced by the interaction effects of dual inoculating bacteria and yeast isolates. SRL has been viewed as a below-ground analog to specific leaf area, where high SRL might facilitate faster growth through more swift resource acquisition. High SRL can enhance nutrient acquisition by permitting the exploration of higher soil volume per unit carbon investment in root length [41]. Since it was found that IAA production and P solubilization increased under co-culturing bacteria with any of the two yeast isolates relative to single culturing, it can therefore be postulated that higher SRL and SVI values (Table 1) were as a result of the interaction between the bacteria and yeast isolates. Furthermore, %YGR and %BGR were highest under T5 and T6; this might also be attributed to the same effects of IAA and P as mentioned above. It is therefore suggested that more research is completed to understand more about the combined influence of IAA and P availability on root parameters such as root branching intensity.

### 3.3. Dual Inoculation of Bacteria and Yeast Enhances Nodule Number per Plant, Nodule Dry Weight, and N-Fixation in Soybean Plants

Results from this study showed significant interaction effects of inoculating bacteria and yeast isolates on NNP, NDW (Table S3), and %Ndfa (Table 2). Soybean root nodules are essential root structures that play a significant role in N_2_-fixation and harboring of plant growth-promoting microbes such as *B. japonicum.* IAA has been reported to play a significant role in nodule development and legume-rhizobia interaction [42,43]. NNP and NDW have also been reported to decrease with the decline in available P [30,31]. This might explain the higher NNP and NDW values under the treatments which had either of the yeast isolate (T3, T4, T5, and T6) relative to T1 and T2 (Table S3). Ahmad et al. [33] also reported enhanced nodulation and nutritional status of mung beans due to co-inoculation of a *Rhizobium* bacteria and P-solubilizing bacteria.

Based on this study’s results, it was found that soybean co-inoculation with *B. japonicum* and *S. cerevisiae* (T5) or *S. exiguus* (T6) enhanced bacterial N_2_-fixation when compared to single bacterial inoculation (T2). This result shows the ability of *S. cerevisiae* and *S. exiguus* to enhance the N_2_-fixation process (Table 2). This might be due to the IAA produced by the two yeast isolates, and also the solubilized P. On the one hand, IAA is a plant growth hormone that has been reported to improve and promote nodule formation and enhance N_2_-fixation in legumes [44,45], induce physiological changes in bacteria such as increased biomass and exopolysaccharide production, as well as infection effectiveness and symbiotic behavior in soybean plants [46]. IAA is also one of the key factors that promote seedling growth, including root growth and nodule development leading to early nodule maturation hence marking the onset of N_2_-fixation [47]. The NNP and NDWP enhancement due to co-inoculation might be caused by the increase in root length and mass, thus increasing the number of effective sites for nodulation and infection sites for the bacteria. On the other hand, when the emphasis is put on the P solubilization abilities of the yeast isolates, soil P availability to soybeans has also been reported to enhance the capacity of root nodules, promoting N_2_-fixation [30,32]. Therefore, the overall impact of the yeast isolates on nodule N_2_-fixation also stems from the importance of P and its effects on host plant growth, nodule formation, nodule growth, nodule function, and nodule metabolism [32]. Several researchers have also corroborated this study’s results of yeast enhancing atmospheric N_2_-fixation when plants are co-inoculated by bacteria and soil yeast. For instance, P deficiency can further reduce bacterial N_2_-fixation in legumes through its basic functions in plants as an energy source in the processes of photosynthesis, translocation of sugars, manufacturing of nucleic acids, glycolysis, membrane production and integrity, respiration, activation or inactivation of enzymes, redox reactions, signaling and carbohydrate metabolism, and other such functions which directly or indirectly influence N_2_-fixation by legume plants such as root and nodule growth [48,49]. Therefore, P is pivotal to nodule morphogenesis and efficient N_2_-fixation.

### 3.4. Bacteria and Yeast Dual Inoculation Shortens the Time Taken to Reach 50% Flowering by Soybean

The productivity of soybeans is largely dependent on the time taken to reach flowering and maturity [50,51]. Higher P availability in the soil leads to the quick flowering of soybeans [51]. In the current study, it was found that soybeans under T3, T4, T5, and T6 took a significantly shorter time to reach 50% flowering relative to treatments T1 and T2 (Figure 3b). It is worth emphasizing that days to reach 50% flowering were shortest under T6 and T5 with the latter having the shortest. This might be because of P solubilization by the yeast isolates, and the increased P solubilization rate when bacteria and yeast isolates were co-inoculated. These results are corroborated by Khan et al. [51] and Tewari. [52] who reported that an increase in available P in the soil results in quick flowering and good flower set in soybeans. There might also be other underlying factors responsible for early flowering in soybeans under microbial inoculation, such as bio fertilizers, that could not be unraveled in this experiment; it is therefore suggested that more research be completed to gain more understanding of the effects of different microbial inoculations on soybean flowering patterns.

### 3.5. Bacteria and Yeast Dual Inoculation on Soybeans Enhances Yield

#### 3.5.1. Root and Shoot Dry Weight

Various studies have revealed that the interaction between bacteria and yeast improves plant biomass [35,53]. Based on our study results, SDW and RDW were significantly higher in soybeans under dual inoculation treatments relative to the rest of the treatments (Figure 4). These results are consistent with those reported by Mohamed [35] and Morsy et al. [53] who also found an increase in soybean dry matter after dual inoculation with bacteria and yeast. This might be because of N_2_-fixation and IAA production by *B. japonicum* [44,54], while yeast isolates solubilize P and also produce IAA [6,35,55,56,57]. As we have mentioned before, IAA is responsible for apical meristematic growth [44], while P plays a crucial role in plant root development [58,59]. These bacteria and yeast capabilities when combined help in shoot and root development by providing more available P and IAA, which combinedly trigger shoot and root growth, hence increasing SDW and RDW. Gaur et al. [60] documented the solubilization of approximately 30–40 kg P_2_O_5_ ha^−1^ due to the inoculation of P-solubilizing microbes. Besides, dual inoculation of beneficial microbes was proven to be more effective than single inoculation considering crop growth and yield because of the augmented effects [34]. Therefore, based on our experiment’s results, we can postulate that dual inoculation of soybean seeds with an N-fixing bacteria and P-solubilizing yeast leads to improved SDW and RDW.

#### 3.5.2. Soybean Seed Yield

The soybean yield is defined as the harvested seed dry matter per unit land area; seed number and PNP are the most important yield components in soybean [61,62]. In our study, we found that SNP and PNP were significantly higher in soybeans under T5 and T6 when compared to the rest of the treatments (Figure 4). Numerous studies have also found that SNP and PNP are significantly enhanced by dual inoculation of IAA-producing and P-solubilizing microbes [34,35]. This highlights the significance of using more than one microbial isolate as a biofertilizer since these isolates can enhance the effectiveness of each other in P solubilization and IAA production as shown in Figure 1. In short, *S. cerevisiae* and *S. exiguous* solubilize P and produce IAA, and *B. japonicum* produces IAA and fixes atmospheric N_2_, but also requires P to drive energy for atmospheric N_2_-fixation and nodule formation [63]. P also provides enough infection sites for *Rhizobium* by promoting root and nodule growth [21]; it assists the N_2_ fixing bacteria in building the mitochondrial and symbiosomal membranes of nodules and assimilation of ammonium as amino acids [64]. Insufficient P encumbers root growth and hampers photosynthesis, as well as the accumulation and translocation of photosynthates and other functions directly related to biological atmospheric N_2_-fixation [34,63]. The scenario of less P and N available or absorbed by the plant, therefore means less plant growth, poor flowering, poor seed set, poor pod filling, and hence low PNP and SNP. 

### 3.6. Dual Inoculation Enhances N and P Accumulation in Soybean Straw and Seed, and Rhizosphere Available P Concentrations

The availability of mineral nutrients is vital for plant growth and survival [65]. High accumulation of P and N in plant biomass including seeds may increase chances of survival through different ways such as faster growth, biotic and abiotic stress tolerance resistance, and successful seed germination. *Rhizobium* and PSB have shown added benefits in legume cultivation by fixing N and solubilizing unavailable P [66]. The existence of these microbes also has some influence on the dynamics between plant and soil rhizosphere available N and P pools. For instance, *Rhizobium* strains fix atmospheric N by forming nodules and contribute to soil fertility and crop yield as biologically fixed N is more sustainable and less prone to volatilization and leaching loss [34,67]. Also, Basu et al. [68] opined that N-fixing bacteria and PSM enhances plant growth and yield by providing nutrients without additional chemical fertilizer input. In our experiment, on the one hand, both straw and seed accumulation of N and P were significantly enhanced by the interaction of *B. japonicum* and *S. cerevisiae* or *S. exiguus* (Table 3). Furthermore, sole bacterial inoculation led to higher N accumulation in both soybean straw and seed relative to sole yeast inoculation, while sole yeast also led to higher P accumulation in both soybean straw and seed relative to sole bacterial inoculation. These results show that *B. japonicum* is efficient in fixing atmospheric N_2_ in soybean, while *S. cerevisiae* and *S. exiguous* are efficient in solubilizing P. These effects, when combined (bacterial-yeast interaction) will therefore lead to higher N and P accumulation in soybean straw and seed. For instance, several studies have shown post-harvest plant sample analyses that are in line with our findings revealing the significant influence of the combined effect of an N-fixing and a P-solubilizing microbe with or without chemical N, and P fertilizers on seed nitrogen, protein, and oil content [69,70] which was also confirmed by Shome et al. [34]. Filipini et al. [71] found and reported higher N concentrations in common beans (*Phaseolus vulgaris*) due to N_2_ fixing microbe inoculation. Furthermore, Estrada-Bonilla et al. [72] reported the presence of higher P concentrations in sugarcane shoots due to the inoculation of P solubilizing microbe compared to non-inoculation. Based on our study results, we can therefore postulate that dual inoculating soybeans with P-solubilizing and N_2_ fixing bacteria enhances N and P accumulation in soybean straw and soybean seeds.

On the other hand, we found that post-harvest rhizosphere soil available N and P were significantly influenced by the interaction of N-fixing bacteria and P-solubilizing yeast (Table S4). For available N, we noticed that concentration levels were lowest in soils under T1, and then followed by T3 and T4. This might be due to the high demand for N by plants under T1 since there is no form of N supplantation, as well as high demand and effective and effective N mineral element scavenging structures (root) due to the abundant P being solubilized by the yeast which enhances root growth. This postulation is corroborated by Liang [73] who mentioned the ability of soil available P to promote root growth. It is worth mentioning that the roots are the plant structures responsible for scavenging and uptake of available N in the rhizosphere [74]. Therefore, higher root growth and density will lead to high N depletion from the rhizosphere [73,74]. Furthermore, the relatively higher available N concentrations in soils under treatment T2 maybe be a result of the BNF by bacteria which led to less extraction of N from the rhizosphere. Based on our results, we found that soil available N concentration values in soils under T5 and T6 were lower than those under T2 but higher than those under T1, T3, and T4. This might be due to the interaction of PSMs and N_2_-fixing bacteria which will lead to an enhanced supply of N to the plants, and P solubilization for plant absorption leading to enhanced root and shoot growth, less demand of N from the rhizosphere, and more available P in the rhizosphere pool. Various authors such as Alori et al. [75], Timofeeva et al. [76], and Bhodiwal et al. [77] have corroborated this; they have reported PSMs to increase available P quantities in the soil. 

Based on our findings, soil available P concentrations were higher in soils under treatment T5 and T6, followed by T3 and T4, then lowest under T1 and T2 (Table S4). This might be due to P solubilization by PSMs under T3 and T4 and enhanced P solubilization under treatment T5 and T6 because the interaction of bacteria and yeast, as mentioned before, increased available P reserves in the rhizosphere. Perhaps the lack of PSMs under treatment T1 and T2 leads to lower available P concentrations after harvesting because the plant was solemnly relying on the P that is already available for plant absorption without supplementation by the means of microbial activity since the soil used was sterilized. These results were also confirmed by Waghmare et al. [69], Jaga et al. [70], and Ahmad et al. [78], who reported soil available P concentrations to be influenced by the presence of PSMs. PSB increases the plant’s P-use efficiency and makes it available in the inorganic form [66]. We, therefore, suggest more research be completed to further unravel potential microbes that can be used for nutrient supplementation during plant growth and also find other materials that can be used to enhance microbial efficiency when used as biofertilizers.

## 4. Materials and Methods

### 4.1. Materials Used and Culture Conditions

The soybean cultivar (Taiwan 292) for the experiment was procured from the Longmen market in Mianyang, China. All microbial inoculants *S. cerevisiae* (PSY-4), *S. exiguous* (strain NCYC 2841)*,* and *B. japonicum* (*B. japonicum* strain TAL-102) were obtained from Beijing Baiou Bowei Biotechnology Limited Company. *S. cerevisiae* was activated on yeast malt agar, pH 6.2 at 26.5 °C [79], *S. exiguus* on malt extract agar, at natural pH and 28 °C [80], and *B. japonicum* on yeast mannitol agar, pH 7.2 at 28 °C [81]. All microbial cultures were stored in 1:2 (*v*:*v*) 50% glycerol at −80 °C for future use. Soybeans were grown in a greenhouse for 3 months. The soybean plants were grown in 10 L plastic pots, each pot was filled with 12 kg sterilized (121 °C for 1 h) soil mix 10:1 (soil and perlite). Distilled water was used for irrigation throughout the soybean cultivation period to constantly keep the soil at field capacity; greenhouse temperatures ranged from 20 to 31 °C and humidity of 55–85% under natural light.

### 4.2. Experimental Design

The pot experiment was a randomized complete block design (RCBD); seed treatment consists of six treatments, i.e., control (T1), *B. japonicum* (T2), *S. cerevisiae* (T3), *S. exiguus* (T4), *B. japonicum × S. cerevisiae* (T5) and *B. japonicum × S. exiguus* (T6). Each treatment was replicated five times. Five pots per treatment per replicate were prepared for sample collection, which means one pot for one of the four selected sets of growing stages were used: cotyledons and unifoliate stage (V_c_), fifth unfolded trifoliate leaves stage (V_5_), early pod fill (R_3.5_) stage, and full maturity stage (R_8_) (refer to Table S1 below for descriptors of soybeans development stages). 

### 4.3. Phosphate Solubilization under In Vitro Conditions

#### 4.3.1. Liquid Culture Media

The ability of the yeast strains to solubilize insoluble P in the liquid was tested by tricalcium phosphate in 100 mL aliquots of Pikovskaya liquid medium [82]. The medium comprised 5 g Ca_3_(PO_4_)_2_, 10 g C_6_H_12_O_6_, 0.2 g KCl_2_, 0.5 g (NH_4_)_2_SO_4_, 0.0001 g FeSO_4_, 0.1 g MgSO_4_·7H_2_O, 0.0001 g MnSO_4_ (pH 6.9). Yeast strains were grown in 100 mL aliquots of the liquid medium for 5 days at 25 °C then the cultures were filtered and centrifuged at 10,000 rpm for 10 min. The supernatant and blank sample pH was determined with the pH meter equipped with a glass electrode. Soluble P in the supernatant and blank sample of the medium were determined using the Molybdenum blue method [83]. 

#### 4.3.2. Soil Culture Media

The ability of the bacterial and yeast strains to solubilize insoluble P in soil was tested by placing 80 g of soil in 9 cm diameter sterile glass Petri dishes. The soil was separately inoculated with 8 mL of each of the different microbial culture combinations suspension (T1 to T6) *B. japonicum* × *S. cerevisiae* and *S. exiguus* (OD_600nm_ 0.8) at the beginning of incubation and with 1 mL of the yeast culture suspension after 7 days. All treatments were replicated five times, and an addition of the second control treatment (T7) was also provided (uninoculated soil with the addition of 2 mL of 0.1 M KH_2_PO_4_·2H_2_O). The addition of an available source of P to the second control treatment was to compare a chemical input with respect to an inoculated strain. Petri dishes were incubated in a moist chamber following a one-week sampling and analysis interval for six weeks; the soil was moistened with sterile deionized water. Soluble P was analyzed following the method by Melich [84].

#### 4.3.3. Determination of the Solubilization Index

The ability of the bacterial and yeast strains to solubilize insoluble P on agar was determined by the solubilization index (SI): the ratio of the total diameter (colony + halo/clear zone) and the colony diameter using Formula (1) [85]. Pikovskaya agar culture media at pH 7, was used. After growing the yeast cells on the medium for 7 days at 25 °C, the halo zone formation around yeast cells was measured.

(1) $$\mathrm{Solubilization}\;\mathrm{index}=\frac{\mathrm{colony}\;\mathrm{diameter}\;+\;\mathrm{halo}\;\mathrm{zone}\;\mathrm{diameter}}{\mathrm{colony}\;\mathrm{diameter}}$$

#### 4.3.4. IAA Production by the Microbial Strains

We assessed the ability of the bacterial and yeast strains to produce a growth-promoting hormone (IAA) by following the method described by Strzelcyk and Pokajska Burdziej [86]. We cultured each of the different microbial strain combinations (T1 to T6) for 5 days at 25 °C in a yeast malt (YM) broth medium. A one hundred milliliter aliquot of the culture filtrate was centrifuged at 1000 rpm for 30 min and the supernatant was acidified to pH 3 with 1N HCl; it was then extracted twice with 100 mL of peroxide-free anesthetic ether in a separatory funnel. The ether extract was then evaporated at 40–45 °C to dryness and the residue was dissolved in 2 mL methyl alcohol and used for determination of IAA. IAA Determination was done by the colorimetric Salkowski reaction [87] in the following way: Two milliliters of the prepared methanolic solution (equivalent to 100 mL of the culture) were added to 4 mL of Salkowski reagent (mixing 2.025 g FeCl_3_, 300 mL H_2_SO_4_ and 500 mL H_2_O). The mixture was kept in the dark for 15–30 min before a colorimetric reading of the developed rosy color using a spectrophotometer (Metash UV-5500PC, Shangai machinery, Shanghai, China) at OD_530nm_. A standard curve of pure grade IAA was constructed. It comprised solutions of different concentrations of a pure IAA in the range 1–10 ppm that were treated with Salkowski reagent (as previously described) and colorimetrically analyzed at OD_530nm_.

#### 4.3.5. Soil Characterization

The soil used in the experiment was collected from the topsoil layer (0–20 cm) in an area that did not have a history of agricultural use (N 31.55340° 104.68249°). We then collected a sample, air dried it, and determined the soil pH following a modified procedure of that outlined by Anderson [88]. The modified procedure includes the use of 10 ± 0.1 g of soil instead of 20 ± 0.1 g of soil and the addition of 25 mL of distilled water as opposed to 50 mL. Air-dried soil was used and prepared in triplicates. All water-soil mixtures were stirred for 10 min on a rotor shaker (SHA-B Laboratory Thermostatic Constant-Temperature Shaker, Changzhou, China) at 250 rpm, then the mixtures were left to stand for 30 min and stirred again for 2 min before measuring the pH using a pH meter (Mettler Toledo 30266628 Fiveeasy Model FP20 Benchtop pH Meter, Columbus, OH, USA). We measured and noted the pH of the supernatant when the reading stabilized (0.1 unit per 30 s or 0.02 units per 5 s) [89]. We extracted available P using the Bray 1 method as described by Bray [90]. One gram of soil sample was placed into a 50 mL tube (conical bottom, screw cap); the extractant was added and the soil solution ratio used was 1:7 (*w*/*v*). Tubes were placed on a reciprocal shaker for 5 min at 200 rpm. After shaking, the soil suspension was centrifuged at 6000 rpm for 5 min and the supernatant was collected. The P concentration of the collected extract (supernatant) was determined colorimetrically by the ascorbic acid method as described by [91]. The N was determined using the Kjeldahl method as described by Bremner and Mulvaney [92]. Soil P, potassium, humus content, electrical conductivity, and textural class were determined using standard procedures as described by Okalebo [93].

#### 4.3.6. Inoculum Preparation and Seed Treatment

To inoculate the seeds of the soybean, we first surface sterilized with 70% ethyl alcohol for 2 min followed by 15 min of soaking in 85% sodium hypochlorite (NaClO), then rinsing 6 times with sterilized distilled water. Peat moss used as an inoculant carrier was pulverized, sieved through a 2 mm (eye diameter) sieve, and sterilized (121 °C for 30 min for three successive days). Peat moss was accordingly neutralized to pH 7 using calcium carbonate (CaCO_3_). Twenty milliliter aliquots of bacteria or yeast broth culture were used per 50 g of the sterilized carrier material; on the scenario of dual inoculation with bacteria and yeast, the single peat inoculants were mixed in equal weights just before seed inoculation. Seeds of each separate treatment were inoculated in polyethylene bags by applying 10 mL of sugar solution. After mixing, the peat inoculant was added and thoroughly mixed with the seeds at a rate of 15 g per 100 g of seeds, and the peat inoculate per gram contained at least 100 viable cells of yeast.

### 4.4. Plant Growth Assessment

#### 4.4.1. Root Elongation Measurements at V_c_

To determine the effect of different microbes and different combinations on early soybean seedling development, the number of germinated seeds was recorded, and the seedling vigor index was calculated at the V_c_ physiological stage as given below [94].

(2) Seedling vigor index=Germination % × (shoot length+root length). 

Fresh roots for the whole plant were carefully harvested, washed, and dried using tissue paper. Roots were carefully laid and scanned, and total root length was measured using ImageJ software (version 1.53t). The percentage yeast-induced root growth response (%YGR), percentage bacterial-induced root growth response (%BGR), and specific root length (SRL) at V_c_ physiological stage were calculated in line with the following formulas by Cloete et al. [95]:

(3) $$\%\mathrm{YGR}=\frac{\mathrm{DW}\;g\;(\mathrm{inoculated})\;-\;\mathrm{DW}\;g\;(\mathrm{control})}{\mathrm{mean}\;g\;\mathrm{DW}\;(\mathrm{control})}\times \;100,$$

where DW indicates the dry weight;

(4) $$\%\mathrm{BGR}=\frac{\mathrm{DW}\;(\mathrm{inoculated})\;(g)\;-\;\mathrm{DW}\;(\mathrm{control})\;(g)}{\mathrm{mean}\;\mathrm{grammes}\;\mathrm{DW}\;(g,\;\mathrm{control})}\times 100,$$

(5) $$\mathrm{SRL}\;[\mathrm{cm}/\mathrm{DW}\;(\mathrm{mg})]=\frac{\mathrm{root}\;\mathrm{length}\;(\mathrm{cm})}{\left[\mathrm{root}\;\mathrm{length}\;\mathrm{DW}\;(\mathrm{mg})\right]}.$$

#### 4.4.2. Nodule Sampling and Determination of Percentage Nitrogen Fixed from the Atmosphere

We collected ten plant samples from each treatment. Collection of samples for fresh and dry weight of shoots and roots/plant was done at V_5_ and R_3.5_ physiological stages. We determined the number of nodules/plant and their dry weights when plants reached the V_5_ and R_3.5_ physiological stages. To do this, samples were carefully removed from the pots and then placed on polyethylene sieves to avoid loss of nodules during washing. The soil was gently washed off the roots under a stream of running tap water and the nodules were carefully removed from the roots and counted. The dry weight was recorded after drying to a constant weight at a temperature of 65 °C.

We then collected the plant sap by root bleeding for the determination of the rate of bacterial N_2_-fixation rate following the micro-scale relative ureide analysis method. Root bleeding xylem sap samples for analysis were collected from each pot when plants reached the V_5_ and R_3.5_ physiological stages. To collect the root-bleeding sap, a very sharp blade was used to cut the shoots just under the cotyledonary node of each plant according to the method described by Peoples et al. [96]. Sap samples were stabilized immediately after collection by mixing with an equal volume of ethyl alcohol in the collection tube and then stored in a freezer. All collected sap samples were kept at −15 °C for long-term storage [96].

Concentrations of ureides (allantoin and allantoic acid) in root bleeding xylem sap were measured as the phenylhydrazone derivative of glyoxylate [97]. Nitrate in xylem sap was determined using the Cu-hydrazine reduction method as described by Kamphake et al. [98]. The amino-N content of sap was determined colorimetrically with ninhydrin, using a 1:1, asparagine: glutamine standard [99,100]. Details of procedures for ureide and amino analysis can be found in Peoples et al. [96].

The percent relative abundance of ureide-N in xylem sap (RUN) was calculated as:

(6) $$\mathrm{RUN}=\frac{400a}{\left(4a+b+c\right)}\times 100,\;$$

where a, b, and c, are, respectively, the molar concentrations of ureides, nitrate, and amino-N [96]. Ureide concentrations, i.e., ‘a’ in Equation (6), were multiplied by four to account for the 4 N atoms in each ureide molecule.

Calculation of %Ndfa (ureide) was based on regressions established from glasshouse calibrations as described by Herridge [101], as follows:

%Ndfa (ureide) = 1.56(RUN − 7.7) (R3.5 = 0.94) vegetative and flowering stages,(7)

%Ndfa (ureide) = 1.56(RUN − 15.9) (R3.5 = 0.94) plants in pod fill.(8)

### 4.5. Plant Material Elemental Analysis

A sample of harvested seeds and straw from 10 treatments was separately collected for elemental and protein content analysis. The N content was determined by firstly wet digesting dry mature seeds or soybean dried straw using the semi-micro Kjeldahl technique followed by distillation and titration [92]. Approximately 0.5 g of plant material was hydrolyzed with 10 mL concentrated sulfuric acid (H_2_SO_4_) accompanied by 0.2 g copper II sulfate (CuSO_4_ 5H_2_O). The mixture was then mounted on a Kjeldahl digestion unit for 60 min at 420 °C. Once digested, the material was diluted to 100 mL using deionized water and then transferred to 100 mL plastic bottles. After cooling, deionized water was added to the hydrolysates before neutralization and titration.

P content was determined following the procedure described by Okalebo et al. [93].

### 4.6. Pod Number per Plant

The seeds were collected at R_8_, the point of harvest (maturation stage). The pod number per plant was obtained at R_6_ by counting the number of pods from 10 plants selected at random in each treatment. The total number of pods was divided by 10 to obtain the average number of pods per plant.

### 4.7. Statistical Analysis

All the data were tested for normality (Shapiro–Wilk test, *p* > 0.05) and homogeneity of variance (Levene’s test, *p* > 0.05); data were transformed (log_10_) if necessary, before two-way ANOVA analyses or one-way ANOVA analysis only for the P solubilization index, P solubilization in both liquid culture media and soil, IAA production by soil yeast strains, and yeast and bacterial count. To fulfill data requirements, the data were tested for differences across treatments; the significance of differences between means was verified using Tukey’s HSD (if the data satisfies the assumption of homogeneity of variance) or Games-Howell post hoc tests (if the Levene’s test for the equality of error variances proved to be statistically significant) [102]. Data analysis was done using the R Language and Environment (version 3.6.3, R Development Core Team 2020) with base packages, OriginPro 2021 (Origin Lab., Northampton, MA, USA), and Inkscape v.1.0 (Draw Freely—Inkscape) to plot the figures.

## 5. Conclusions

Dual inoculation of N_2_-fixing bacteria (*B. japonicum*) and P-solubilizing yeast (*S. cerevisiae* or *S. exiguous*), improved soybean germination, P solubilization, IAA production, nodulation, atmospheric N_2_-fixation, growth, flowering, yield, and N and P accumulation. Maximum, P solubilization, IAA production, N_2_-fixation, plant growth, flowering, seed yield, and N and P accumulation were found under dual inoculation of “(*B. japonicum* × *S. exiguous*) and (*B. japonicum × S. cerevisiae*)” with the latter prevailing over the other under all categories. Based on our study results, we can therefore conclude that the dual inoculation of *B. japonicum* and *S. cerevisiae* or *S. exiguus* has an augmented effect on soybean germination, nodulation, BNF fixation, root and shoot growth, flowering, nutrient accumulation, and seed yield. The synergy between soil PSM and N-fixing microbial strains nodulating leguminous plants represents a key rhizosphere process that deserves to be thoroughly investigated to improve rhizosphere biological functions in grain legumes and enhance the sustainability of legume-based cropping systems. In line with this, several studies reported that soil application of PSM, including PSB, individually or in a consortium, increased plant growth through solubilization of insoluble P and subsequent increase in BNF. Therefore, the application of N-fixing bacteria and PSM in nutrient-deficient soils, where large amounts of mineral nutrients are fixed in unavailable forms, could help farmers increase crop yields and financial returns via more rational use of P fertilizer inputs and an increase in BNF. Therefore, we recommended that farmers consider combining nitrifying and mineral P-solubilizing microbes to their soybean cropping systems to increase their harvest and income level. The replacement of mineral nitrogen fertilizers with microbial fertilizers is well-justified from the perspective of sustainable crop production and the production of high-quality organic food.

## Figures and Tables

**Table 1:** Differences in the seedling vigor index, specific root length, and germination percentage of soybean at V_c_ physiological stages.

Treatment	Seedling Vigor Index	Specific Root Length (cm mg^−1^)	Germination Percentage (%)
T1	17.9 ^d^ ± 0.20	0.03 ^a^ ± 0	97.5 ^a^ ± 0.3
T2	22.9 ^c^ ± 0.18	0.03 ^a^ ± 0	97.7 ^a^ ± 0.2
T3	24.6 ^b^ ± 0.26	0.03 ^a^ ± 0	97.7 ^a^ ± 0.2
T4	24.5 ^b^ ± 0.20	0.03 ^a^ ± 0	98 ^a^ ± 0.1
T5	32.2 ^a^ ± 0.35	0.03 ^a^ ± 0	98 ^a^ ± 0.1
T6	31.5 ^a^ ± 0.71	0.03 ^a^ ± 0	97.7 ^a^ ± 0.2
Bacteria	**	ns	ns
Yeast	***	ns	ns
Bacteria × yeast	****	ns	ns

Data (means ± SE, *n* = 5). *p*-values of two-way ANOVAs followed by different superscripts (a–d) denote significant differences between treatments at *p* < 0.05 according to Tukey’s HSD. *p*-values of two-way ANOVAs of bacteria, yeast, and their interaction (bacteria × yeast) are indicated: *p* < 0.05, *; *p* < 0.01, **; *p* < 0.001, ***; *p* < 0.0001, ****; ns, not significant. Treatments: non-inoculated soybeans (T1), soybean inoculated with *B. japonicum* (T2), *S. cerevisiae* (T3), *S. exiguus* (T4), *B. japonicum* × *S. cerevisiae* (T5) or *B. japonicum* × *S. exiguus* (T6).

**Table 2:** Differences in relative ureide-N (%) (RUN %) and percentage of N derived from the atmosphere (%Ndfa), at V_5_ and R_3.5_ physiological stages across treatments.

Treatment	RUN%	RUN%	%Ndfa	%Ndfa
	V_5_	R_3.5_	V_5_	R_3.5_
T1	5.4 ^c^ ± 0.91	9.4 ^c^ ± 0.33	−3.6 ^c^ ± 1.42	−10.2 ^c^ ± 1.52
T2	50.1 ^b^ ± 2.50	62.3 ^b^ ± 0.86	66.2 ^b^ ± 3.90	72.4 ^b^ ± 1.34
T3	6.8 ^c^ ± 0.40	11.3 ^c^ ± 1.19	0 ^c^ ± 0.63	−7.3 ^c^ ± 1.85
T4	6.5 ^c^ ± 0.90	10.3 ^c^ ± 1.49	−2 ^c^ ± 1.40	−8.8 ^c^ ± 2.33
T5	61.3 ^a^ ± 2.21	77.8 ^a^ ± 1.91	83.7 ^a^ ± 3.44	90.7 ^a^ ± 1.29
T6	58.2 ^a^ ± 1.85	75 ^a^ ± 0.98	78.8 ^a^ ± 2.88	88.3 ^a^ ± 2.13
Bacteria	****	****	****	****
Yeast	ns	ns	ns	ns
Bacteria × yeast	****	****	****	****

Data (means ± SE, *n* = 5). *p*-values of two-way ANOVAs followed by different letters denote significant differences between treatments at *p* < 0.05 according to Tukey’s HSD. *p*-values of two-way ANOVAs of bacteria, yeast, and their interaction (bacteria × yeast) are indicated: *p* < 0.05, *; *p* < 0.01, **; *p* < 0.001, ***; *p* < 0.0001, ****; ns, not significant. Treatments: non-inoculated soybeans (T1), soybean inoculated with *B. japonicum* (T2), *S. cerevisiae* (T3), *S. exiguus* (T4), *B. japonicum* × *S. cerevisiae* (T5) or *B. japonicum* × *S. exiguus* (T6).

**Table 3:** Differences in soybean straw N, Straw P, seed N concentration, and seed P concentration across treatments at R8 physiological stage, and rhizosphere available P after the experiment.

Treatment	Straw N Concentration (mg g^−1^)	Straw P Concentration (mg g^−1^)	Seed Nitrogen Concentration (mg g^−1^)	Seed P Concentration (mg g^−1^)
T1	28.51 ^d^ ± 3.41	72.91 ^d^ ± 3.21	50.57 ^d^ ± 3.43	102.91 ^d^ ± 3.21
T2	58.73 ^b^ ± 2.16	80.78 ^c^ ± 2.16	75.83 ^b^ ± 6.15	114.78 ^c^ ± 2.16
T3	38.06 ^c^ ± 2.03	97.23 ^b^ ± 4.38	64.77 ^c^ ± 3.55	123.23 ^b^ ± 4.38
T4	37.26 ^c^ ± 2.63	95.08 ^b^ ± 3.21	63.42 ^c^ ± 3.12	121.08 ^b^ ± 3.21
T5	69.18 ^a^ ± 2.48	121.66 ^a^ ± 5.59	85.32 ^a^ ± 1.84	151.66 ^a^ ± 5.59
T6	67.24 ^a^ ± 1.97	128.36 ^a^ ± 4.81	84.98 ^a^ ± 1.17	148.36 ^a^ ± 4.81
Bacteria	***	*	****	*
Yeast	*	**	***	***
Bacteria × yeast	****	****	****	****

Data (means ± SE, *n* = 5). *p*-values of two-way ANOVAs followed by different letters denote significant differences between treatments at *p* < 0.05 according to Tukey’s HSD. *p*-values of two-way ANOVAs of bacteria, yeast, and their interaction (bacteria × yeast) are indicated: *p* < 0.05, *; *p* < 0.01, **; *p* < 0.001, ***; *p* < 0.0001, ****; ns, not significant. Treatments: non-inoculated soybeans (T1), soybean inoculated with *B. japonicum* (T2), *S. cerevisiae* (T3), *S. exiguus* (T4), *B. japonicum* × *S. cerevisiae* (T5) or *B. japonicum* × *S. exiguus* (T6).

## Data Availability

Not applicable.

## Supplementary Materials

The following supporting information can be downloaded at: https://www.mdpi.com/article/10.3390/plants12030681/s1, Figure S1: In vitro determination of antagonism between bacteria and different yeast isolates, and P solubilization after culturing microbes for 7 days on Pikovskaya agar medium showing the clear zone of phosphate dissolution; Figure S2: Invitro determination of IAA production in liquid media by different microbes; Figure S3: Five-day-old soybean seedlings germinated under different microbial inoculation treatments; Table S1: Descriptors of soybean development stages; Table S2: Physiochemical characteristics of a representative composite soil sample used; Table S3: Differences in nodule number per plant and nodule dry weight per plant of soybean at V_5_ and R_3.5_ physiological stages under different treatments; Differences in the rhizosphere soil N and P concentrations under different treatments. Stages under different treatments; Table S4: Differences in the rhizosphere soil N and P concentrations under different treatments.
