# Supplementary material for: Effects of Co-Inoculating Saccharomyces spp. with Bradyrhizobium japonicum on Atmospheric Nitrogen Fixation in Soybeans (Glycine max (L.))
Source: Plants (Basel). 2023 Feb 3;12(3):681. doi: 10.3390/plants12030681 (PMC9919766; doi:10.3390/plants12030681)
Supplement: Supplementary file 1 [file plants-12-00681-s001.zip › plants-2183531-supplementary.pdf]

Supplementary information

Table S1. Descriptors of soybeans development stages [99,100].

Physiological stage	Description
$V_{(n)}$	Vegetative phases of growth, with (n) denoting the number of nodes on the main stem
R_1	Early flowering
$R_{3.5}$	Late flowering
R_3	Early pod-fill; very small pods
$R_{4\text{-}5}$	Mid-pod-fill; larger pods with beans starting to develop
R_6	Late pod-fill; pods containing full-sized green beans
R_7	Physiological maturity; pods yellowing and about 50% leaves yellow
R_8	Harvest maturity; pods brown

Table S2. Physiochemical characteristics of a representative composite soil sample used.

Properties	Value
Sand (%)	36.9±0.6
Silt (%)	25.9±0.4
Clay (%)	37.7±0.9
Texture grade	Clay loam
Humus (%)	0.63±0.003
pH	6.3±0.05
EC (dsm^{-1})	0.41±0.002
Available nitrogen (mg kg^{-1})	730±0.5
Available phosphorus (mg kg^{-1})	2.43±0.005
Potassium (mg kg^{-1})	500±2.23

Table S3. Differences in nodule number per plant and nodule dry weight per plant of soybean at V_5 and $R_{3.5}$ physiological stages under different treatments.

Treatments	NNP		NDWP (mg $plant^{-1}$)	
	V_5	$R_{3.5}$	V_5	$R_{3.5}$
T1	13.25^{d}±1.49	14.50^{d}±1.0	210.50^{d}±22.92	201.00^{d}±52.39
T2	26.50^{b}±0.87	33.00^{b}±1.55	430.50^{b}±14.05	536.25^{b}±19.20
T3	21.25^{c}±1.03	28.50^{c}±1.55	344.50^{c}±16.54	496.50^{c}±13.32
T4	19.50^{c}±1.04	27.75^{c}±1.85	336.25^{c}±16.85	468.50^{c}±13.91
T5	36.75^{a}±1.11	78.55^{a}±2.55	591.25^{a}±18.10	1277.25^{a}±94.90
T6	33.75^{a}±0.75	74.30^{a}±4.5	580.73^{a}±12.14	1259.25^{a}±98.37
Bacteria	***	****	****	****
Yeast	**	***	***	****
Bacteria × yeast	****	****	****	****

*Data (means ± SE, n = 5). P-values of two-way ANOVAs followed by different superscripts denotes significant differences between treatments at p < 0.05 according to Tukey's HSD. P-values of two-way ANOVAs of bacterial, yeast, and their interaction (bacteria × yeast) are indicated p < 0.05, *; p < 0.01, **; p < 0.001, ***; p < 0.0001, ****; ns, not significant. Treatments: non-inoculated soybeans (T1), soybean inoculated with B. japonicum (T2), S. cerevisiae (T3), S. exiguus (T4), B. japonicum × S. cerevisiae (T5) or B. japonicum × S. exiguus (T6).*

Table S4. Differences in the rhizosphere soil N and P concentrations under different treatments. stages under different treatments.

Treatments	Rhizosphere soil available N concentration (mg g^{-1})	Rhizosphere soil available P concentration (mg g^{-1})
T1	5.52±0.03^{d}	1.86±0.01^{c}
T2	6.21±0.05^{a}	1.73±0.03^{c}
T3	5.33±0.04^{c}	2.96±0.03^{b}
T4	5.31±0.03^{c}	2.94±0.03^{b}
T5	5.73±0.04^{b}	3.21±0.02^{a}
T6	5.73±0.04^{b}	3.2±0.02^{a}
Bacteria	***	*
Yeast	**	****
Bacteria × yeast	*	****

*Data (means ± SE, n = 5). P-values of two-way ANOVAs followed by different superscripts denotes significant differences between treatments at p < 0.05 according to Tukey's HSD. P-values of two-way ANOVAs of bacterial, yeast, and their interaction (bacteria × yeast) are indicated p < 0.05, *; p < 0.01, **; p < 0.001, ***; p < 0.0001, ****; ns, not significant. Treatments: non-inoculated soybeans (T1), soybean inoculated with B. japonicum (T2), S. cerevisiae (T3), S. exiguus (T4), B. japonicum × S. cerevisiae (T5) or B. japonicum × S. exiguus (T6).*

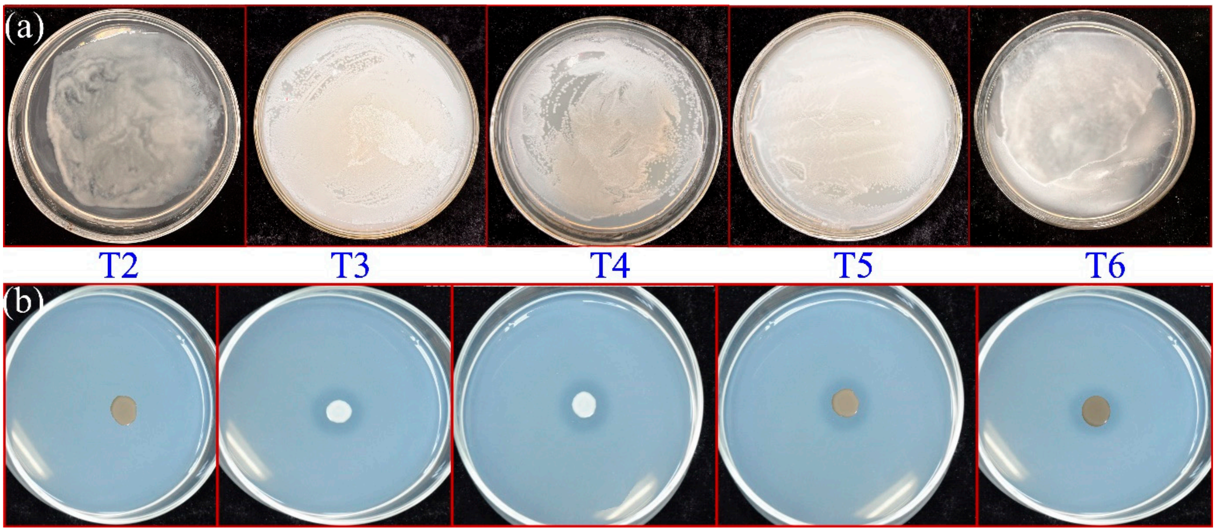

Figure S1. Invitro determination of (a) antagonism between bacteria and different yeast isolates, and (b) P solubilization after culturing microbes for 7 days on Pikovskaya's agar medium showing the clear zone of phosphate dissolution. Treatments: non-inoculated soybeans (T1), soybean inoculated with B. japonicum (T2), S. cerevisiae (T3), S. exiguus (T4), B. japonicum × S. cerevisiae (T5) or B. japonicum × S. exiguus (T6).

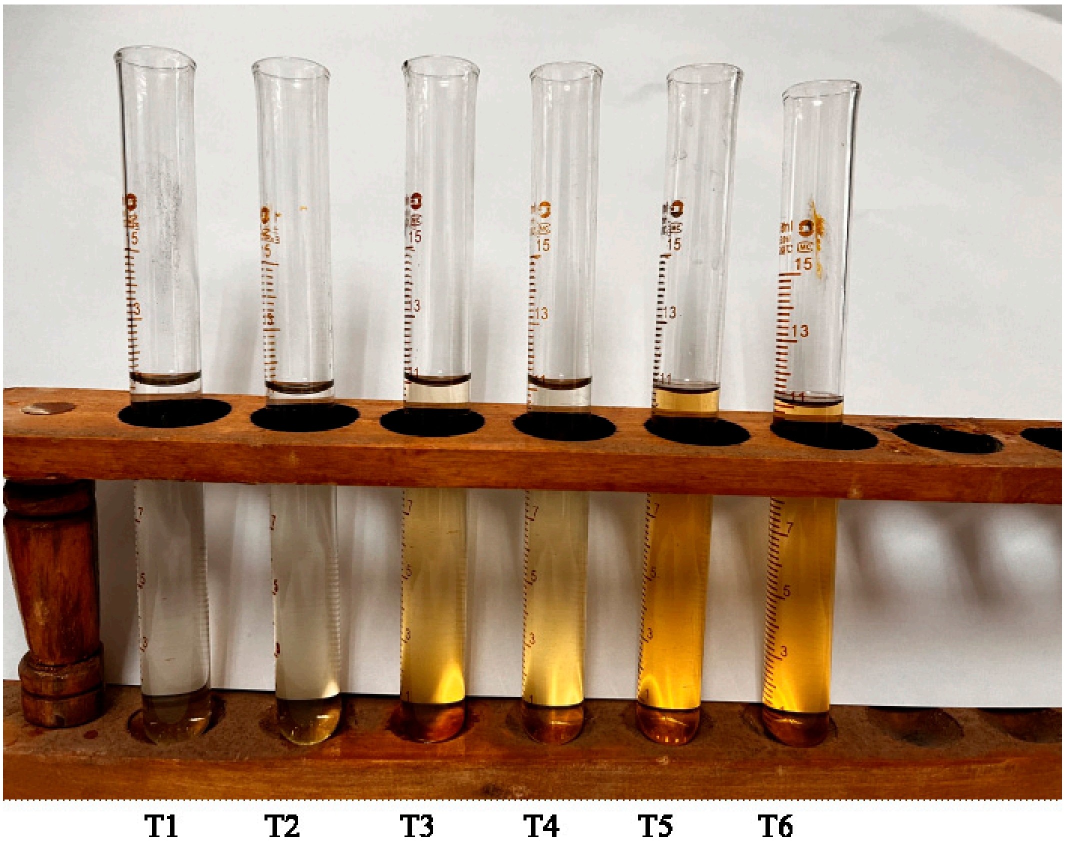

Figure S2. Invitro determination of IAA production in liquid media by different microbes. Treatments: non-inoculated soybeans (T1), soybean inoculated with B. japonicum (T2), S. cerevisiae (T3), S. exiguus (T4), B. japonicum × S. cerevisiae (T5) or B. japonicum × S. exiguus (T6).

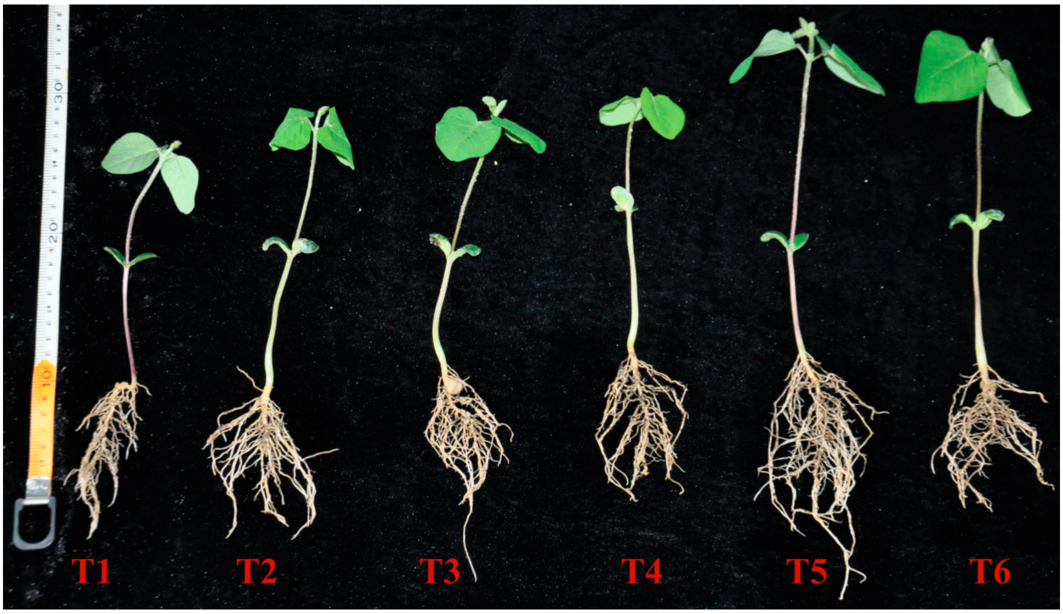

Figure S3. Five-day old soybean seedlings germinated under different microbial inoculation treatments. Treatments: non-inoculated soybeans (T1), soybean inoculated with B. japonicum (T2), S. cerevisiae (T3), S. exiguus (T4), B. japonicum × S. cerevisiae (T5) or B. japonicum × S. exiguus (T6).
